# The Efficacy of Ramucirumab in the Treatment of Gastric or Gastroesophageal Junction Cancer: A Meta-Analysis of RCTs

**DOI:** 10.1155/2021/8960315

**Published:** 2021-02-22

**Authors:** Hongqiong Yang, Yaojun Zhou, Liangzhi Wang, Tianyi Gu, Mengjia Lv, Jinling Sun, Chao Tu, Junbo He

**Affiliations:** ^1^Department of General Practice, The Third Affiliated Hospital of Soochow University, Changzhou, Jiangsu, China; ^2^Department of Surgical Urology, The Third Affiliated Hospital of Soochow University, Changzhou, Jiangsu, China

## Abstract

Five electronic databases were searched for eligible records. Outcomes were presented and analyzed according to the objective response rate (ORR), progression-free survival (PFS) rate, and overall survival (OS) rate. Five records involving 2,024 participants were included in the study. The pooled analysis of OS and PFS were longer with ramucirumab (RAM) therapy than without RAM for OS (odds ratio (OR) = 0.90, 95% confidence interval (CI) = 0.82–1.00, *p* = 0.05) and PFS (OR = 0.74, 95%CI = 0.57–0.96, *p* = 0.02). Moreover, compared with the current first-line chemotherapy, the OS (OR = 0.93, 95%CI = 0.83–1.04, *p* = 0.19) and PFS (OR = 0.82, 95%CI = 0.64–1.06, *p* = 0.13) results were not significantly higher with RAM. The ORRs of the patients in the RAM therapy groups were significantly higher than those in the groups without RAM (OR = 1.40, 95%CI = 1.14–1.73, *p* = 0.001).

## 1. Introduction

Gastric/gastroesophageal junction cancer (GC/GEJC) is known to be one of the leading causes of cancer-related death worldwide [1, 2]. The first-line treatment for GC/GEJC is a combination medication of platinum-based and fluoropyrimidine-based therapy [3]. Unfortunately, when the first-line treatment fails to control the disease, there are few options left for patients. Therefore, there is an urgent need for new systemic targeted agents of GC/GEJC to be developed.

Vascular endothelial growth factor (VEGF) and the VEGF receptor-2 (VEGFR-2) signaling pathway and angiogenesis procedure play critical roles in the pathogenesis of gastric cancer. The tumoral VEGF concentrations are reported to be significantly related to increased tumor aggressiveness and worse survival in patients with GC/GEJC [4, 5]. In a preclinical study, the application of the VEGFR-2 targeted antibody proved effective in inhibiting tumor-induced angiogenesis [6]. A lot of VEGFR-2-targeted antibodies have been developed as a potential therapeutic approach to GC/GEJC [7]. Among them, ramucirumab (RAM) is a fully human immunoglobulin G-1 (IgG1) monoclonal VEGFR-2-targeted antibody, which prevents ligand binding and receptor-mediated pathway activation in endothelial cells [8]. It has been launched in clinical trials to treat various cancers, such as colorectal carcinoma [9], hepatocellular carcinoma [10], urothelial carcinoma [11], non-small-cell lung cancer [12], and GC/GEJC [13–17]. Many reports have analyzed the safety of RAM as a mono-/add-on therapy [18–23]. Some studies have considered the efficacy of RAM in the treatment of solid tumors [24–26]. However, few studies have summarized the efficacy of RAM in GC/GEJC treatment [27]. In this study, we conducted a meta-analysis of the efficacy of RAM in the treatment of GC/GEJC based on five randomized controlled trials (RCTs).

## 2. Materials and Methods

This meta-analysis was conducted in accordance with the Preferred Reporting Items for Systematic Reviews and Meta-Analyses (PRISMA) [28].

### 2.1. Literature Search

PubMed, Embase, the Cochrane Library, Web of Science, and ClinicalTrials.gov were searched to obtain relevant records with no language restrictions (published up to May 2020). The following MeSH terms were used as subject strategies: (“Gastric Cancer” OR “Stomach Cancer” OR “GC” OR “Gastro-oesophageal” OR “gastroesophageal junction cancer” OR “GEJ” AND “ramucirumab”).

### 2.2. Inclusion Criteria

Studies were included in the meta-analysis if they met the following criteria: (1) the studies were designed as RCTs, (2) patients were treated using ramucirumab alone or plus chemotherapy versus placebo or chemotherapy alone, (3) patients were clinically diagnosed with G/GEJ cancer, (4) the outcome of interest was the efficacy of the treatment, and (5) a full-text paper was available.

### 2.3. Risk of Bias Assessment

The risk of bias in the literature was evaluated by two independent investigators. The study quality was justified using the Cochrane Collaboration's “Risk of Bias” tool.

### 2.4. Data Extraction

Two researchers extracted the contents from each included trial independently. Any disagreement on the data extraction was resolved by discussion or consultation with a third investigator. The main categories were extracted based on the following information: lead author, publication year, treatment regimen, patient number, age, sex number, and outcome measures.

### 2.5. Quality Assessment

Two researchers evaluated the risk of bias of the included clinical trials independently using the Cochrane Collaboration Tool [29]. The following quality assessments were included: bias from selection, attrition, performance, reporting, detection, and other items. The results were classified as “low risk,” “high risk,” and “unclear risk” according to the Cochrane instructions. A consensus from all group members was reached through a discussion when discrepancies were found.

### 2.6. Statistical Analysis

The therapeutic efficacy of ramucirumab on GC/GEJC patients was assessed by computing the objective response rate (ORR), pooled overall survival (OS) rate, and progression-free survival (PFS) rate, along with their 95% confidence intervals (CIs) extracted from the selected literature. The statistical heterogeneity of the studies was verified by the chi-squared test and *I*^2^ statistic. A random-effects model was used when statistically significant heterogeneity was identified (*I*^2^ > 50% or *p* value < 0.1 indicated high heterogeneity) [30]; otherwise, we chose the fixed-effects Mantel–Haenszel model. All statistical calculations were performed using STATA version 15.0 (STATA Corp., College Station, TX) and Review Manager version 5.3 (Nordic Figure Cochrane, Copenhagen, Denmark). The reliability of the results was tested by sensitivity analysis through omitting individual studies. Begg's and Egger's tests were performed to assess publication bias [31, 32].

## 3. Results

### 3.1. Study Selection and Characteristics

**Figure 1:**
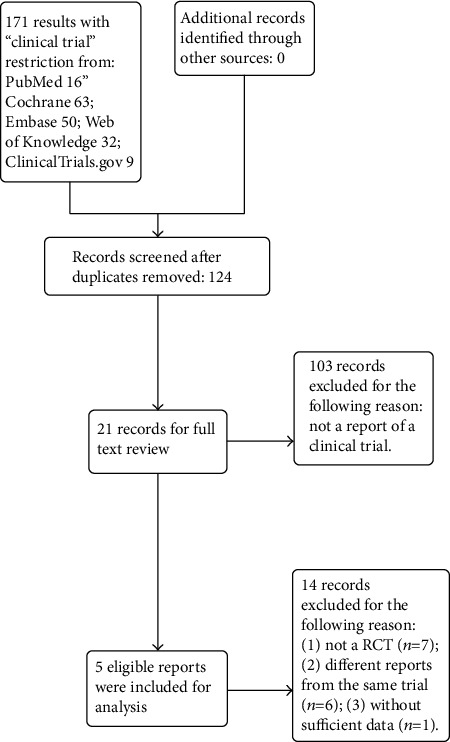
Flow chart for selection of relevant studies.

A total of 124 studies were retrieved after the removal of the duplicated reports. Following this, 107 irrelevant citations were removed because they were not clinical trials. Twenty-one reports were left for full-text review. Among them, seven trials were not RCTs, six articles were subanalyses of previous trials, and one did not have sufficient data. Therefore, a final total of five RCTs [13–17] were selected for this meta-analysis. The search process is described in Figure 1, and the basic characteristics of the eligible trials are listed in Table 1.

### 3.2. Efficacy Outcomes of the Therapy

#### 3.2.1. Overall Survival

**Figure 2:**
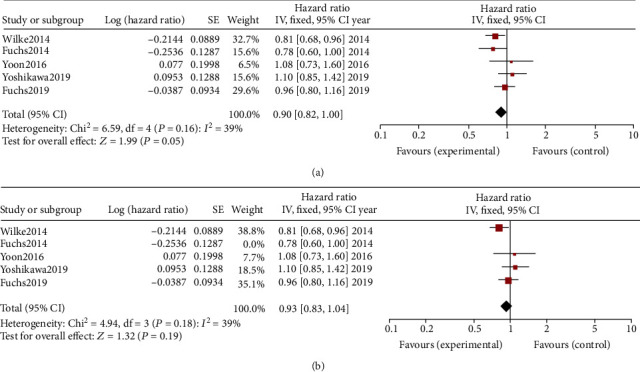
(a) Pooled analysis of overall survival (OS) comparing the application of ramucirumab (RAM) alone or plus chemotherapy with the control (placebo or chemotherapy). (b) Pooled analysis of OS comparing the application of RAM alone or plus chemotherapy with chemotherapy alone.

Pooled analysis of OS comparing the application of RAM alone or plus chemotherapy with the control (placebo or chemotherapy) is shown with a forest plot chart (Figure 2). Pooling the OS demonstrated that the application of RAM led to an OS advantage (Figure 2(a), OR = 0.90, 95%CI = 0.82–1.00, *p* = 0.05). However, a subgroup analysis comparing the RAM therapy including/excluding a certain kind of chemotherapy with chemotherapy alone did not indicate a significantly superior OS result (Figure 2(b), OR = 0.93, 95%CI = 0.83–1.04, *p* = 0.19). In Yoon et al.'s [15] phase II clinical trial, RAM+mFOLFOX6 therapy did not result in a significantly better OS outcome in patients with advanced esophageal, gastroesophageal junction, or gastric adenocarcinoma. The median OS of the RAM+mFOLFOX6 arm was 11.7 months (95%CI = 10.2–14.6) and the mFOLFOX6 arm was 11.5 months (95%CI = 9.0–15.3). Similar results were found in Yoshikawa et al.'s [17] phase II trial, where the median OS of the RAM+S-1+oxaliplatin group was 14.65 months (95%CI = 12.39–15.67) and the median OS of the placebo+S-1+oxaliplatin group was 14.26 months (95%CI = 13.83–17.31).

#### 3.2.2. Progression-Free Survival

**Figure 3:**
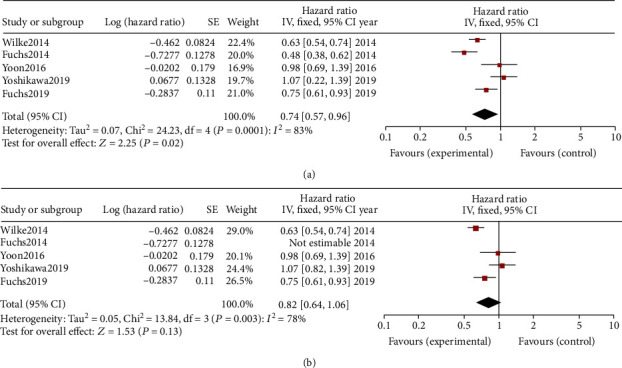
(a) Pooled analysis of progression-free survival (PFS) comparing the application of ramucirumab (RAM) alone or plus chemotherapy with the control (placebo or chemotherapy). (b) Pooled analysis of PFS comparing the application of RAM alone or plus chemotherapy with chemotherapy alone.

Pooled analysis of progression-free survival (PFS) comparing the application of RAM alone or plus chemotherapy with the control (placebo or chemotherapy) was shown with a forest plot chart (Figure 3). Pooled estimates of effect sizes showed that the difference of PFS between the two groups was statistically significant (Figure 3(a), OR = 0.74, 95%CI = 0.57–0.96, *p* = 0.02). A random-effects model was used for the analysis due to significant heterogeneity (*I*^2^ = 83%, *p* < 0.0001). Subgroup analysis comparing the RAM therapy with/without a certain kind of chemotherapy with chemotherapy alone did not indicate a significantly superior PFS result (Figure 3(b), OR = 0.82, 95%CI = 0.64–1.06, *p* = 0.13). Again, a random-effects model was used for the analysis due to significant heterogeneity (*I*^2^ = 78%, *p* = 0.003). In Yoshikawa et al.'s [17] phase II trial, the median PFS of the RAM+S-1+oxaliplatin group was 6.34 months (95%CI = 5.65–6.93) and the median PFS of the placebo+S-1+7.13 group was 6.72 months (95%CI = 5.75–7.13).

#### 3.2.3. Objective Response Rate

**Figure 4:**
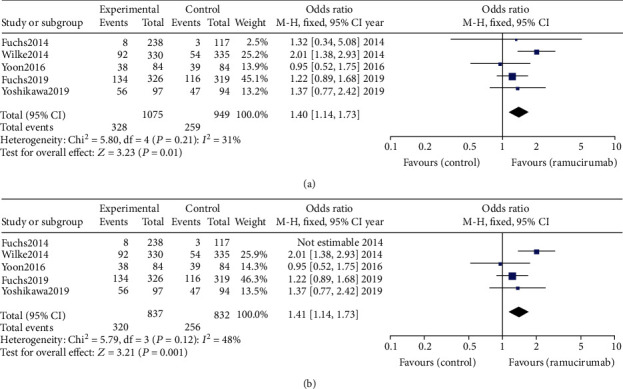
(a) Pooled analysis of objective response rate (ORR) comparing the application of ramucirumab (RAM) alone or plus chemotherapy with the control (placebo or chemotherapy). (b) Pooled analysis of ORR comparing the application of RAM alone or plus chemotherapy with chemotherapy.

The ORR was defined as the percentage of participants who achieved the best overall response of partial response (PR) or complete response (CR). A significantly better ORR was detected in groups with RAM therapy compared with the control (Figure 4(a); OR = 1.40, 95%CI = 1.14–1.73, *p* = 0.001). Pooled analysis of ORR comparing the RAM plus a certain kind of chemotherapy with chemotherapy alone also led to a better ORR outcome (Figure 4(b); OR = 1.41, 95%CI = 1.14–1.73, *p* = 0.001). In Yoon et al.'s [15] trial, the RAM+mFOLFOX6 treatment did not exceed in ORR (45.2, 95%CI = 34.3–56.5%) compared with mFOLFOX6 therapy alone (46.4%, 95%CI = 35.5–57.6%).

### 3.3. Publication Bias and Sensitivity Analysis

**Figure 5:**
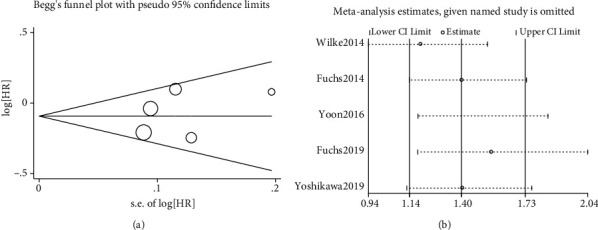
(a) Begg's funnel plot of publication bias. (b) Sensitivity analysis of the pooled objective response rate (ORR).

Begg's and Egger's tests were used to verify the publication bias of the included studies. Begg's test result was *z* = 0.73 and *p* = 0.462, and Egger's test result was *t* = 0.62 and *p* = 0.580. A funnel plot of Begg's test is shown in Figure 5(a). In brief, Begg's test and Egger's test results indicated no significant publication bias in this meta-analysis. A sensitivity analysis was conducted to test the stability of our evaluation. The results indicated that no individual study had consequential effects among the included studies (Figure 5(b)). The results of the current research were credible and stable.

### 3.4. Assessment of Study Quality

**Figure 6:**
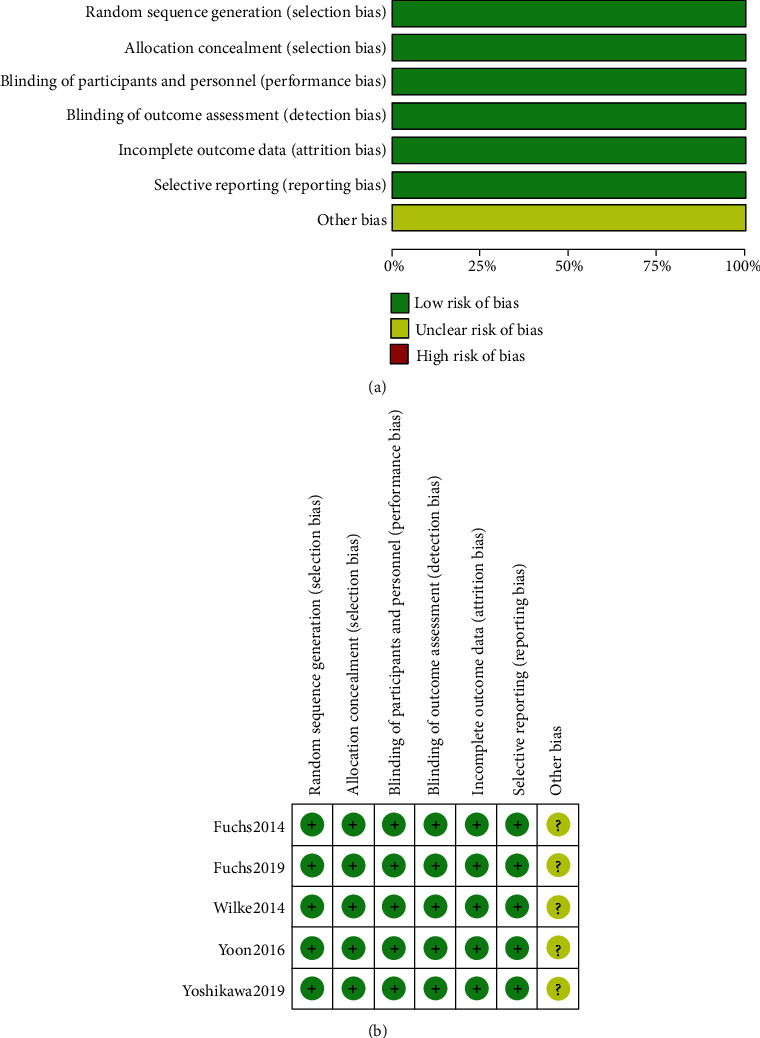
(a) Risk of bias graph. (b) Risk of bias summary.

The quality of the selected trials was assessed and reported by Review Manager v5.3. The results showed that the overall risk of bias is low; the risk of bias graph and summary are shown in Figure 6. In summary, the quality of the studies met the criterion.

## 4. Discussion

Although the current standard chemotherapy or novel biologic agents have been largely used, the advanced stage GC/GEJC patients have poor clinical outcomes. Abundant studies have focused on evaluating the combinations of immune checkpoint inhibitors, standard chemotherapy, and biological ligands to prolong patients' survival and improve their quality of life. Unfortunately, most chemotherapy approaches exhibit unsatisfactory performance when it comes to providing substantial medication and life benefits [33]. Our meta-analysis depicts the evidence on RAM, a VEGFR-2-targeted antibody, for advanced GC/GEJC treatment in clinical practice.

Pooled analysis by Li et al. [34] indicated that the expression of VEGFR-2 is a predictor of gastric cancer survival. Patients with overexpression of VEGFR-2 had a significantly increased risk of median OS. The inhibition of VEGFR-2 is expected to be great potential therapy for the treatment of GC/GEJC. Findings from our meta-analysis suggested that compared with the current standard chemotherapy, the RAM treatment as add-on therapy did not significantly improve the OS (OR = 0.93, 95%CI = 0.83–1.04, *p* = 0.19) or PFS (OR = 0.82, 95%CI = 0.64–1.06, *p* = 0.13). As for the overall survival of the GC/GEJC patients, in Yoon et al. [15] and Yoshikawa et al. [17], the application of RAM to mFOLFOX6 combination and S-1+oxaliplatin did not significantly extend the patients' median OS (Table 1). However, positive results in terms of a prolonged median OS were shown when RAM was used as add-on therapy to PTX and cisplatin+capecitabine, respectively (Table 1). Similar phenomena were found in the patients' median PFS in Yoon et al. [15] and Yoshikawa et al. [17], where the median PFS of the RAM add-on group (Yoon et al., 11.7 months, 95%CI = 10.2–14.6; Yoshikawa et al., 14.65 months, 95%CI = 12.39–15.67) was not significantly improved compared with that of the control group (Yoon et al., 11.5 months, 95%CI = 9.0–15.3; Yoshikawa et al., 14.26 months, 95%CI = 13.83–17.31). There is a report about VEGFR-2 and the hepatocyte growth factor receptor (MET) having a synthetic effect on tumor growth [35]. The inhibition of VEGFR-2 prohibits angiogenesis and attenuates tumor growth, but cancers may bypass this effect through the activation of MET. This may provide an explanation for why the RAM add-on therapy did not provide significantly prolonged median OS and PFS rates in some clinical trials. Zhang et al. [36] demonstrated that the inhibition of both VEGFR-2 and MET yielded a more promising effect on suppressing tumor growth and metastasis in hepatocellular carcinoma than blocking VEGFR-2 did. This may provide evidence for further therapy approaches for the combination medication of VEGFR-2 and MET inhibition.

The patients' ORR in the RAM add-on group was significantly higher than it was in the chemotherapy group (OR = 1.41, 95%CI = 1.14–1.73, *p* = 0.001). In Fuchs et al.'s [13] phase III trial, where RAM was used as a monotherapy, the ORR of both groups was extremely low (RAM, 3.4%, 95%CI = 1.5–6.5; placebo, 2.6%, 95%CI = 0.5–7.3). This suggests that RAM as a monotherapy is not an efficient medication for advanced GC/GEJC treatment, although the ORR was slightly higher than that in the placebo group in Fuchs et al.'s [13] research. In Yoon et al.'s [15] study, RAM did not enhance the ORR of the GC/GEJC patients (RAM+mFOLFOX6, 3.4%, 95%CI = 1.5–6.5). The precise explanation for this phenomenon remains to be explored. The data provide critical information for the future treatment of GC/GEJC with RAM. The present study found that the combination medication of RAM+S-1+oxaliplatin resulted in the best outcome in the five included RCTs.

## 5. Conclusion

In conclusion, the results indicated that RAM exhibits effectual antitumor activity in GC/GEJC patients compared with placebo and some first-line treatments, such as PTX and cisplatin+capecitabine, regarding the OS, PFS, and ORR. However, compared with S-1+oxaliplatin and mFOLFOX therapy, RAM did not exhibit superior efficacy in terms of OS or PFS. Our findings suggest that RAM is not a game changer in GC/GEJC therapy.

## Figures and Tables

**Table 1:** Basic characteristics of the included studies.

Study	ClinicalTrials.gov (number)	Phase	*N*	Treatment setting	Line of therapy	Median OS (months + 95% CI)	ORR (%)
Fuchs 2014 (REGARD)	NCT00917384	III	238	8 mg/kg RAM IV+BSC	2^nd^ line	5.2 (4.4 to 5.7)	3.4 (1.5 to 6.5)
			117	Placebo+BSC		3.8 (2.8 to 4.7)	2.6 (0.5 to 7.3)
Wilke2014 (RAINBOW)	NCT01170663	III	330	8 mg/kg RAM IV + 80 mg/m^2^ PTX	2^nd^ line	9.6 (8.5 to 10.8)	27.9 (23.3 to 33.0)
			335	Placebo + 80 mg/m^2^ PTX		7.4 (6.3 to 8.4)	16.1 (12.6 to 20.4)
Yoon 2016	NCT01246960	II	84	8 mg/kg RAM IV+mFOLFOX6	1^st^ line	11.7 (10.2 to 14.6)	45.2 (34.3 to 56.5)
			84	Placebo+mFOLFOX6		11.5 (9.0 to 15.3)	46.4 (35.5 to 57.6)
Yoshikawa 2019 (RAINSTORM)	NCT02539225	II	97	8 mg/kg RAM IV + 80-120 mg/m^2^ S-1 + 100 mg/m^2^ oxaliplatin	1^st^ line	14.65 (12.39 to 15.67)	58.2 (49.7 to 66.7)
			94	Placebo + 80-120 mg/m^2^ S-1 + 100 mg/m^2^ oxaliplatin		14.26 (13.83 to 17.31)	50.0 (41.3 to 58.7)
Fuchs 2019 (RAINFALL)	NCT02314117	III	326	8 mg/kg RAM IV + 80 mg/m^2^ cisplatin IV + 1000 mg/m^2^ capecitabine	1^st^ line	11.17 (9.92 to 11.93)	41.1 (35.8 to 46.4)
			319	Placebo + 80 mg/m^2^ cisplatin IV + 1000 mg/m^2^ capecitabine		10.74 (9.53 to 11.89)	36.4 (31.1 to 41.6)

ORR: objective response rate; OS: overall survival; RAM: ramucirumab; BSC: best supportive care; PTX: paclitaxel; mFOLFOX6: administered IV per manufacturer's instructions for each drug substance on day 1 of each cycle (14 days/cycle). Oxaliplatin 85 mg/m^2^, leucovorin 400 mg/m^2^, 5-fluorouracil (5-FU) 400 mg/m^2^ bolus 5-FU 2400 mg/m^2^, 5-FU 2400 mg/m^2^ continuously given over 46–48 h; S-1: tegafur-gimeracil-oteracil potassium.

## Data Availability

The datasets generated for this study are available from the corresponding author upon reasonable request.
